# Strain-induced metal–semiconductor transition observed in atomic carbon chains

**DOI:** 10.1038/ncomms7636

**Published:** 2015-03-30

**Authors:** A. La Torre, A. Botello-Mendez, W. Baaziz, J. -C. Charlier, F. Banhart

**Affiliations:** 1 Institut de Physique et Chimie des Matériaux de Strasbourg, UMR 7504 CNRS, Université de Strasbourg, 23 rue du Loess, 67034 Strasbourg, France; 2 Institute of Condensed Matter and Nanosciences, Université catholique de Louvain, Chemin des étoiles 8, 1348 Louvain-la-Neuve, Belgium; 3 Institut de Chimie et Procédés pour l’Energie, l’Environnement et la Santé, UMR 7515 CNRS Université de Strasbourg, 25 rue Becquerel, 67087 Strasbourg, France

## Abstract

Carbyne, the *sp*^1^-hybridized phase of carbon, is still a missing link in the family of carbon allotropes. While the bulk phases of carbyne remain elusive, the elementary constituents, that is, linear chains of carbon atoms, have already been observed using the electron microscope. Isolated atomic chains are highly interesting one-dimensional conductors that have stimulated considerable theoretical work. Experimental information, however, is still very limited. Here we show electrical measurements and first-principles transport calculations on monoatomic carbon chains. When the 1D system is under strain, the chains are semiconducting corresponding to the polyyne structure with alternating bond lengths. Conversely, when the chain is unstrained, the ohmic behaviour of metallic cumulene with uniform bond lengths is observed. This confirms the recent prediction of a metal–insulator transition that is induced by strain. The key role of the contacting leads explains the rectifying behaviour measured in monoatomic carbon chains in a nonsymmetric contact configuration.

## Abstract (web summary)

Previous studies have predicted that carbyne (a single chain of *sp* ^1^ carbon atoms) goes from being metallic to semiconducting under strain. Here, the authors measure the electronic properties of carbyne, confirming the prediction and showing that the conductivity is determined by strain and by the contacts.

## Graphical abstract

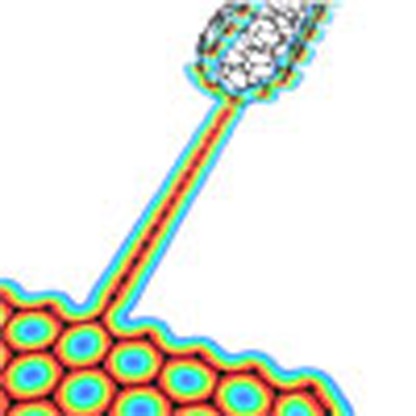

Linear chains of carbon atoms are the textbook example for *sp*^1^-hybridized carbon. The bulk modification, known as carbyne or linear acetylenic carbon^1^, is considered as an arrangement of linear chains and has been subject of many efforts in synthetic chemistry^2^. However, the existence of this exotic phase of carbon has remained the subject of debates until today. It is generally assumed that carbyne is unstable because of the exothermic formation of cross-links between neighbouring chains of atoms^3^. Isolated chains, however, have been observed in several electron microscopy studies in recent years and appear to be stable^4^,^5^,^6^,^7^,^8^,^9^,^10^. Interest in them has increased because of predictions concerning their unusual and, in many respects, extreme mechanical and electronic properties^11^,^12^.

Two configurations have been proposed for carbyne as a one-dimensional (1D) crystal, namely *cumulene* with double bonds over the whole chain (=C=C=C=C=C=) and *polyyne* with alternating single and triple bonds (−C≡C−C≡C−C≡). In the symmetric cumulene structure, the π-electrons are uniformly distributed over the chain, while they are mostly localized at the triple bonds in the broken-symmetry polyyne. This makes cumulene a metallic conductor, while polyyne is expected to be semiconducting. As a 1D electron system, cumulene can be destabilized by Peierls distortion so that polyyne with alternating bond lengths becomes energetically favoured^13^,^14^. The Peierls distortion increases with increasing strain, as it has been predicted in recent studies^12^,^15^,^16^, leading to an increasing bandgap. However, a recent theoretical consideration suggested that the energetic gain by the Peierls instability in unstrained chains is so small that it can be overcompensated by zero-point vibrations^16^. This is a most important result since it predicts that metallic cumulene exists and should prevail in mechanically relaxed chains. At increasing strain, however, the Peierls dimerization dominates and leads to the transformation to polyyne chains and the opening of a bandgap.

Although many theoretical studies of carbon chains have been published in the past decade, the experimental information has remained very limited. This is because of the difficulty to produce and characterize these highly reactive rows of atoms. Besides the existence of carbon chains, no experimental proof for any of their exciting properties has been given until a recent study has shown the first measurements of the electrical conductivity^15^. The conductivity is much lower than expected, and indications for semiconducting behaviour appear in the first current–voltage curves. Strain in the chain has been proposed as the major factor that determines the bandgap of the chain.

Here it is shown experimentally that unstrained chains are metallic, while strained chains exhibit a distinct semiconducting behaviour. This confirms the recent prediction of a strain-induced metal–semiconductor transition in carbyne^16^. In addition, first-principles calculations suggest that the hybridization state of carbon atoms at the end of the chain has a decisive influence on the transport behaviour of the system and explains the low conductivity. Furthermore, rectifying behaviour in the electrical conductivity is observed when the contacts are different.

## Results

### Strained carbon chains

The experiments were carried out in a dedicated specimen stage in a transmission electron microscope. Graphitic aggregates were contacted by a metallic tip^17^ and retracted while an electrical current flowed through the system. In such a way, chains of carbon atoms were unravelled from the graphitic material. While the chains bridged the gap between the contacts, their electrical properties were measured. Current–voltage curves allow to determine under what conditions the chains are metallic or semiconducting, although the bandgap itself cannot be quantified (three- or four-point measurements would be necessary, which is presently impossible on these elusive objects). While linear curves (ohmic behaviour) are expected for metallic systems, S-shaped curves should occur for semiconductors with two end contacts of the same type.

**Figure 1:**
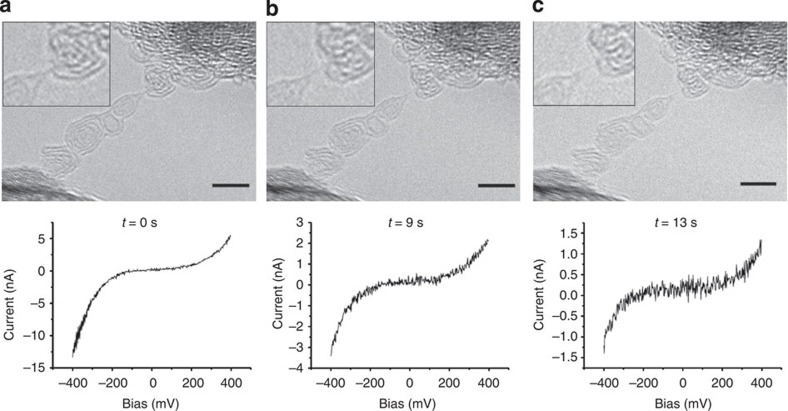
Transmission electron microscopy (TEM) images and corresponding current–voltage curves of a strained carbon chain. The temporal evolution of a carbon chain between two graphitic contacts is shown; (**a**) at an arbitrary time zero; (**b**) after 9 s; (**c**) after 13 s. See also the Supplementary Movie. The corresponding current–voltage curves illustrate qualitatively the same semiconducting behaviour, although with a decreasing conductivity from **a** to **c**. Scale bars, 2 nm.

Figure 1 shows the evolution of a chain between two graphitic contacts (the sequence is also shown in the Supplementary Movie). The length of the chain increases slightly because of an ongoing unravelling. The S-shaped current–voltage curves are a clear signature for semiconducting behaviour. A slight asymmetry is visible, which points to a small difference in the electronic structure of the two contacts. However, what is evident though is a clear decrease in the current as the chain evolves. Within the ±400-mV sweep of the bias, the current varies over a range of ~18, 5 and 2 nA, respectively, in the three measurements. This is in accordance with a previous calculation^15^, showing that the conductivity decreases considerably with increasing length of a semiconducting chain in the presence of strain. This eventually leads to an opening of the bandgap.

**Figure 2:**
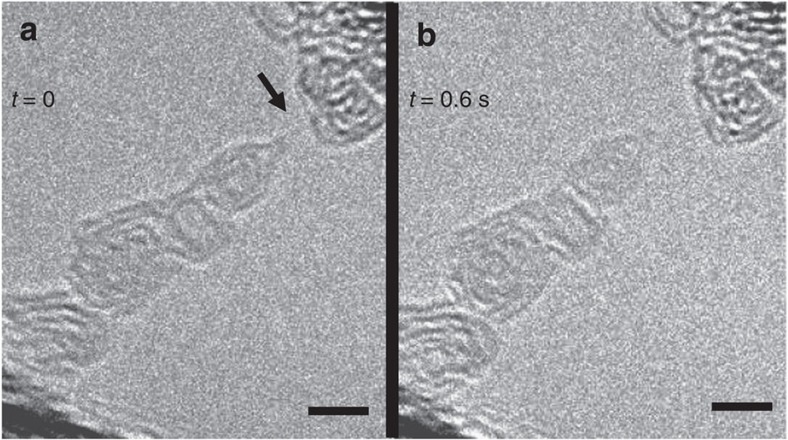
Rupture of a carbon chain. (**a**) shows the chain (arrowed) of Fig. 1c where a new time zero is defined. (**b**) shows the situation after 0.6 s; the chain has ruptured. The relaxation of the bottom contact towards the upper left corner of the image in **b** demonstrates that the chain has been under mechanical stress. Scale bar, 2 nm.

To shed more light on the question of strain, Fig. 2 shows the rupture of the chain after the measurements of Fig. 1 (see Supplementary Video 1). The relaxation of the graphitic aggregate towards the left hand side after the breakage of the chain shows that the arrangement has been under stress before the chain ruptured. Therefore, the chain must have been in a strained state while the measurements have been taken. This confirms the presence of semiconducting polyyne in the strained case. A direct measurement of the forces in the chain was not possible with the present set-up.

**Figure 3:**
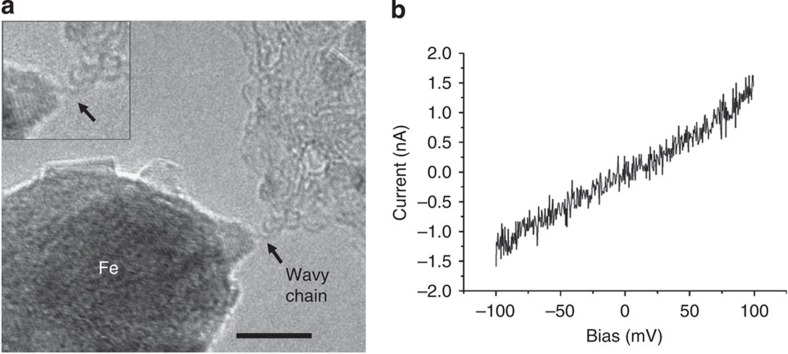
Wavy carbon chain. (**a**) shows the TEM image and (**b**) the corresponding current–voltage curve. The wavy shape indicates vanishing strain. The current–voltage curve is linear, corresponding to ohmic conductivity (see also Fig. 6). Scale bar, 5 nm.

An unstrained chain is shown in Fig. 3. Apparently, the two contacts approached each other after the formation of the chain, leading to the wavy shape. The current–voltage curve shows a linear behaviour. Such an ohmic characteristic is in accordance with the prediction of metallic *cumulene* under a very small strain (less than 3% but also depending on temperature)^16^.

### The influence of contacts

**Figure 4:**
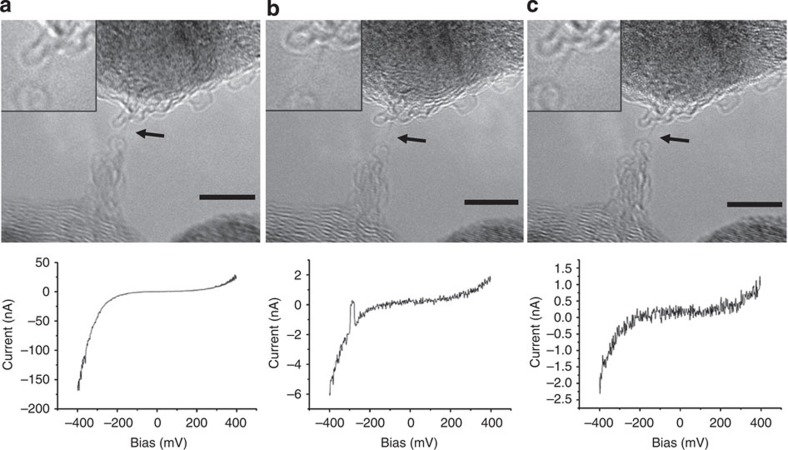
Carbon chain between two different contacts. The temporal evolution is shown: (**a**) *t*=0; (**b**) *t*=6 s; (**c**) *t*=16 s. The current–voltage curves show an asymmetric shape, which is typical for rectifying behaviour. (The hump in the middle curve is because of an instability of the contacts, while the *I/V* curve has been recorded). Scale bar, 5 nm.

**Figure 5:**
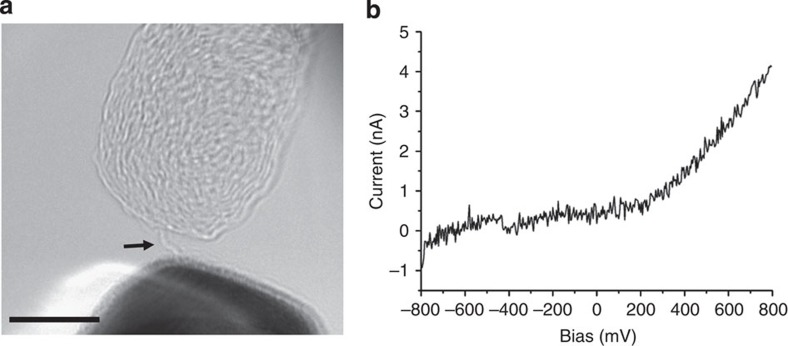
Carbon chain between two dissimilar electrodes. The bottom electrode is Fe; the top electrode carbon. (**a**) TEM image; (**b**) *I/V* curve. The current–voltage curve is asymmetric. Scale bar, 5 nm.

The type of the contact turned out to be another factor that has an important influence on the current–voltage characteristics of the chains. Figure 4 shows a chain that is connected at its bottom side to a round graphitic structure that is most likely the closed end of a single-wall nanotube. The top contact is difficult to specify but could be either a piece of graphene or the Fe crystal above (overlap in the projected image does not allow to follow the chain to its end). At first, the same behaviour as for the chain in Fig. 1 is observed, namely a progressive decrease in conductivity. However, the current–voltage characteristic is clearly asymmetric. Such a behaviour is typical for a diode that could be of the Schottky type. This is where the Fermi energy of the metal (upper conduction band edge) is not at the same level as the bottom conduction band edge of the semiconductor. This behaviour is confirmed in Fig. 5 where the bottom electrode is clearly a Fe crystal (the chain is slightly out of focus).

**Figure 6:**
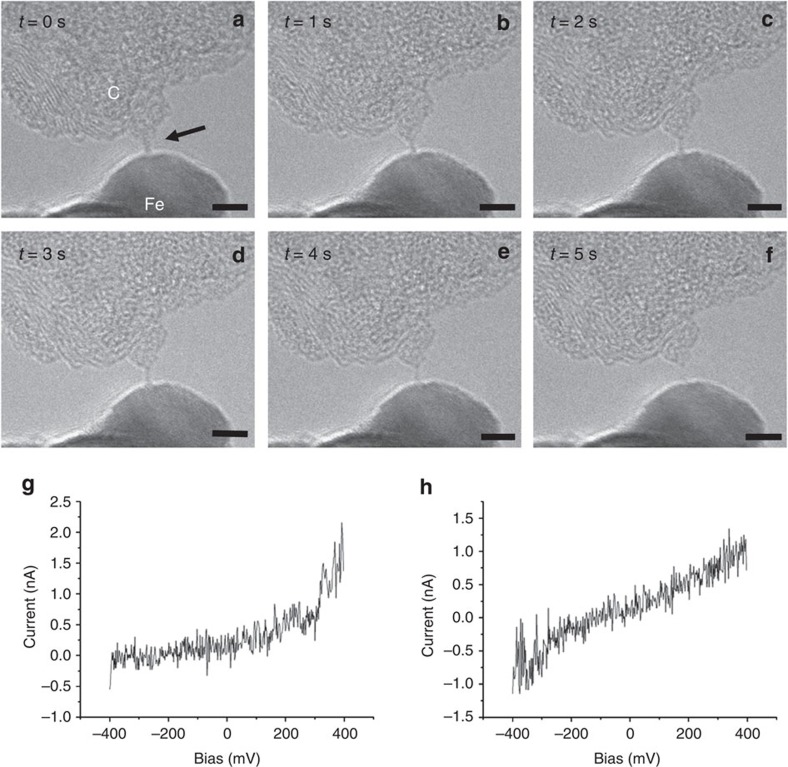
Carbon chain between a Fe contact and a graphitic aggregate. TEM images (**a**–**f**) at different times (indicated) are shown. While the images do not show a visible change in morphology, two current–voltage curves (**g**,**h**) taken at different times during the recording of the image series show rectifying (**g**) versus ohmic (**h**) behaviour. This can be explained by a sudden release of strain due to a slight movement of one of the electrodes, leading to a semiconductor–metal transition in the chain. Scale bar, 5 nm.

Another example of a metal contact on one side and a carbon contact on the other is shown in Fig. 6. Here the chain does not visibly change its morphology; however, one current–voltage curve shows the rectifying behaviour while a second one taken within the lifetime of the chain is ohmic. This shows that the *I/V* curves can change their characteristics, although the contact geometry is unchanged. It can be explained by a sudden release of strain due to a slight movement of one of the electrodes, leading to a semiconductor–metal transition in the chain. In contrast to the unstrained chain in Fig. 2 that shows a buckled appearance, the present chain remains straight, and it is not just the shape of the chain that determines the *I/V* curve.

## Discussion

The temperature of the chains is a parameter that has to be considered in view of the predicted strain–temperature diagram of carbyne^16^. The diagram indicates a certain influence of temperature on the phase transition between *cumulene* and *polyyne*. At room temperature, the transition should occur at 2–3% strain, whereas only 1% is sufficient at 500 °C. The temperature of the chain in the present experiment cannot be measured but it can be expected that ohmic heating is small, since the current in the chain remains in most cases below 10 nA at applied voltages up to 400 mV (heating by the electron beam is always negligible). The chains are short and their thermal conductivity should be extremely high; therefore, a rapid dissipation of ohmic heat towards the contacts should prevent considerable heating. Several theoretical studies predict that the electrical conductivity of chains with an even number of atoms is higher than of odd-number chains^11^,^18^,^19^. However, indications for these conductivity oscillations, when the chain is getting longer or shorter, have not been seen in this study. Nor do changes in the direction of rectification appear, as it has been predicted for the switching between even and odd number of atoms^20^.

Recent simulations of the anharmonic quantum vibrational structure of carbyne predict that zero-point atomic vibrations eliminate the Peierls distortion in the unstrained chain, preserving the *cumulene* symmetry^16^. Consequently, when the chain is not stressed, its metallic character is conserved, thus explaining (as mentioned above) the ohmic behaviour measured for wavy chains. On the other hand, when strain is applied to the chain, a semiconducting behaviour is observed, leading to an important decrease in the conductivity as the length of the chains increases^15^. This behaviour is observable in Figs 1 and 4.

**Figure 7:**
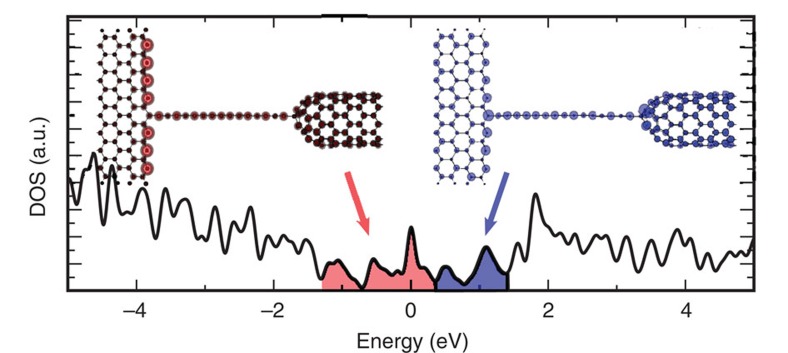
First-principles density of states of a carbon chain. The chain is connected to two different types of contacts: *sp*^2^ (graphene nanoribbon) and *sp*^3^ (carbon nanotube). The projection on real space of the shaded red and blue regions of the DOS is illustrated on the nanoribbon–chain–nanotube system, respectively. The charge neutrality point (Fermi energy) of the system is the reference zero energy.

However, such a strain-induced modification of the electronic structure in the chain cannot explain on its own the low conductivity values recorded in the measurements. Indeed, the nature and the characteristics of the contacts at the graphitic periphery play a key role—both in the conduction process and in the rectifying behaviour. In order to investigate transport properties in a connected chain, quantum conductance calculations were conducted using the Landauer–Büttiker approach^21^ and compared with the experimental measurements. Within such a framework, a carbon chain of *ca.* 2 nm is placed between two semi-infinite leads. Previous calculations have well characterized the change of current through the chain, as a function of length, showing that it decreases exponentially^15^. In order to mimic as close as possible the graphitic periphery, which connects the chain in the experiment, two different contacts were envisaged: a graphene nanoribbon (with a *sp*^2^ connection to the chain) on one side and a capped carbon nanotube (with a *sp*^3^ connection to the chain) on the other side (see Fig. 7). This system generalizes the different contact possibilities (for example, to a graphene flake, a metallic particle or graphite-covered metallic particle) while keeping the main characteristics of the observed phenomena, that is, the contact asymmetry. The atomic structure of the nanoribbon–chain–nanotube arrangement has been relaxed and optimized, using *ab initio* techniques^22^. The chain segment is no longer a perfect 1D crystal, but it preserves the bond alternation characteristic of the *polyyne* configuration (~1.32 and ~1.38 Å).

The density of states (DOS) of the nonsymmetric nanoribbon–chain–nanotube system is also calculated and exhibits many sharp peaks (Fig. 7), which can be attributed to localized states coming from the bare zigzag edge of the nanoribbons and the pentagons, which are incorporated in the cap of the nanotube^23^.

**Figure 8:**
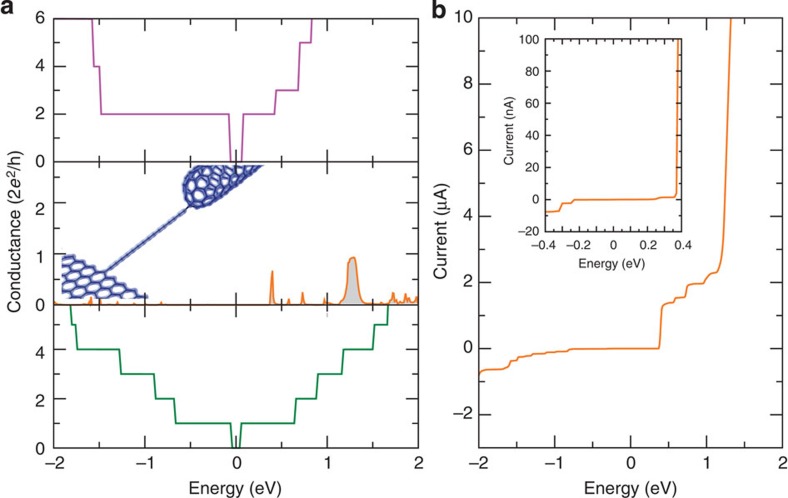
Quantum conductance calculations. (**a**) shows the transmission along an infinite nanotube (purple), an infinite nanoribbon (green) and through the carbon chain contacted to a semi-infinite nanoribbon on one side and a semi-infinite capped nanotube on the other side (orange). The middle panel also includes the *ab inito* electronic charge density of the nanoribbon–chain–nanotube configuration. (**b**) Calculated current–voltage curve for the nanoribbon–chain–nanotube system exhibiting a clear rectifying behaviour. The inset presents a close-up in a narrower window of energy.

The electronic states below and close to the Fermi energy (*E*_F_=0 eV) are found to be mainly localized in the central region. Indeed, the spatial contribution of the red-shaded region of the DOS is concentrated around the bare zigzag edges of the ribbon, the chain and the nanotube cap, as illustrated in Fig. 7. In contrast, the electronic states of energy larger than +0.4 eV (blue-shaded region of Fig. 7) are spread all over the nanoribbon–chain–nanotube system. The electronic charge density along both the chain and the leads is illustrated in Fig. 8a (middle panel). It presents a clear depletion of the π-electron cloud close to the chain–nanotube contact (*sp*^3^ connection—see arrow), which is not present at the other side (nanoribbon with the *sp*^2^ connection).

Within this theoretical approach, the quantum conductance of the carbon chain can be estimated. The transmission function is computed using the Landauer–Büttiker formalism and the surface Green’s function matching method, after extracting the first-principles Hamiltonian and overlap matrices^24^,^25^. This conductance is presented in Fig. 8a for an infinite nanotube (top), an infinite nanoribbon (bottom) and through the nanoribbon–chain–nanotube system (middle). In contrast to both the ribbon and the tube, the electronic transmission through the chain is the signature of a resonant tunnelling transport. Indeed, only a few channels at specific energy windows allow the electrons to travel between the two graphene/nanotube-based reservoirs. The corresponding *I/V* curves are presented in Fig. 8b, depicting an asymmetry between positive and negative bias voltage. A drastic increase in the current appears to be ~0.4 eV, in good agreement with the measurements (see inset in Fig. 8b), and 1.2 eV. Such a rectifying behaviour can be attributed to the different nature of the two *sp*^2^/*sp*^3^ contacts at the chain (see Fig. 8a—middle panel). A large plateau of quasi-zero conductivity is observed between −1.2 and +0.4 eV, although electronic states are present in the DOS within this energy window (Fig. 7—red-shaded area). However, as already mentioned above, these electronic states are mainly localized in the central region and do not contribute to the conductance. In contrast, the states of energy larger than +0.4 eV (Fig. 7—blue-shaded region) are spread all over the system, thus allowing electronic transport. Nevertheless, the low conductivity between −1.2 and +0.4 eV matches the experimental measurements quite well and explains why the overall conductivity of a carbon chain between realistic contacts is clearly lower than predicted in previous calculations for idealized chains^11^,^26^.

It is important to note that, although the specific example of a chain in between a nanoribbon and a nanotube is used, each with specific dimensions, the explanation of the rectification behaviour is general. Namely, that it is a tunnelling resonant process in which the three systems (the semi-infinite left conductor, the semi-infinite right conductor and the chain coupled to the two systems) have to exhibit channels at the same energy in order for the electrons to go from one conductor to the other, albeit scattered by interference and reflections.

In conclusion, the recent prediction of metal–insulator transition in atomic carbon chains^16^ is shown experimentally for the first time. Whereas zero strain leads to cumulene with ohmic behaviour, the semiconducting characteristics of polyyne appear in a strained chain. The contacts are found to decisively determine the electrical properties of the system. With realistic contacts, resonant tunnelling and the existence of few energy channels do not allow the previously predicted large currents through the chains at low bias.

## Methods

### Experiments

Carbon chains were produced by contacting graphitic aggregates with a metallic scanning tunnelling microscopy (STM) tip, passing a current through the contact, and slowly retracting the tip. This can lead to an unravelling of carbon chains from graphene layers or nanotube-like structures^15^. The procedure of tip fabrication and the contact chemistry have been described in recent studies^17^. The experiment was carried out in the specimen stage of a transmission electron microscope (Jeol 2100 F with C_s_-corrected condenser, located at the Institut de Physique et Chimie des Matériaux de Strasbourg (IPCMS) in Strasbourg). A Nanofactory holder, containing an STM tip, was used to contact the specimen and to measure the electrical conductivity of the objects. This allowed the observation of the formation of carbon chains at high image resolution and the measurement of the electrical properties at the same time. Once the formation of a chain was seen during the retraction of the tip from the graphitic electrode, electrical measurements were carried out.

The presence of chains cannot always be deduced without any doubt from the images. This is because of the appearance of narrow graphene ribbons or thin nanotube-like objects between the electrodes and slight vibrations of the objects. However, every chain that could be identified clearly showed a much lower conductivity than even the smallest ribbons or tubes. Within the typical voltage sweep of ±100–400 mV, currents of several hundreds of nanoamperes or even microamperes were measured in all ribbons and tubes while chains typically showed less than 10 nA. This could be used as a proof that objects with a slightly unclear appearance in the image were chains and not, for example, ribbons in side-view.

### Computations

Density functional theory (DFT) computations were performed using the SIESTA code^22^ with a double-zeta polarized basis set, GGA (PBEsol) functional and Troullier–Martins pseudopotentials in a mesh equivalent to an energy cutoff of 500 Ry. Atomic structures were allowed to relax until forces were reduced to within 0.02 eV Å^−1^. For the computation of the self-consistent Hamiltonian of the semi-infinite leads, periodic boundary conditions and 30 k-points in the periodic direction were used. A gamma point calculation, comprising the nanotube–chain–nanoribbon system with three unit cells as buffer layer for the nanoribbon and nanotube was used to generate the Green’s function where the interaction with the leads was introduced as self-energies according to the surface matching method^24^.

## Author contributions

A.L.T. and A.B.-M. equally contributed to this work: A.L.T. conceived and carried out the experimental measurements and A.B.-M. designed and implemented the simulation model. W.B. synthesized the starting material. J.C.-C. and F.B. elaborated the scientific basis and supervised the study. All authors discussed the results and wrote the manuscript.

## Additional information

**How to cite this article**: La Torre, A. *et al*. Strain-induced metal–semiconductor transition observed in atomic carbon chains. *Nat. Commun.* 6:6636 doi: 10.1038/ncomms7636 (2015).

## Supplementary Material

Supplementary Movie 1 The movie shows, in real time, the evolution of the atomic carbon chain shown in Figures 1 and 2 and the final rupture.
